# Smooth muscle contractile responses to bile acids in mouse ileum require TGR5 but not ASBT

**DOI:** 10.3389/fneur.2024.1334319

**Published:** 2024-04-24

**Authors:** Diana S. Chang, Krishnakant G. Soni, Geoffrey A. Preidis

**Affiliations:** Division of Gastroenterology, Hepatology & Nutrition, Department of Pediatrics, Baylor College of Medicine and Texas Children’s Hospital, Houston, TX, United States

**Keywords:** sex differences, ursodeoxycholic acid, deoxycholic acid, apical sodium-dependent bile acid transporter, Takeda G protein-coupled receptor 5

## Abstract

**Background:**

Many disorders of gut-brain interaction (DGBIs) are more prevalent in women than men and feature alterations in gastrointestinal motility and bile acid homeostasis. Mechanisms by which bile acids regulate gastrointestinal motility are poorly characterized. We recently validated an adapted tissue bath technique using everted mouse ileum, which revealed differential contractile responses to ursodeoxycholic acid (UDCA) and deoxycholic acid (DCA). Here, we aimed to determine whether these responses are dependent on host sex, the plasma membrane bile acid receptor TGR5, or the apical sodium-dependent bile acid transporter ASBT.

**Methods:**

Ileal segments from male and female mice were everted and suspended in tissue baths. Contractile responses to physiologic concentrations of UDCA and DCA were quantified with or without TGR5 or ASBT inhibitors. Phosphorylation of extracellular signal-regulated kinase (ERK) and myosin light chain (MLC), markers of TGR5 activation and smooth muscle contraction, respectively, were assessed with western blot.

**Results:**

There were no sex differences in the dose-dependent contractile responses to bile acids. At 100 μmol/L, UDCA but not DCA increased MLC phosphorylation and increased contractility. TGR5 inhibition decreased ERK phosphorylation and led to decreases in contractility, phosphorylated MLC, and surprisingly, total MLC. ASBT inhibition did not affect contractile responses.

**Conclusion:**

Differential effects of UDCA and DCA on ileal smooth muscle contractility are not dependent on host sex or ASBT-mediated transport. Bile acids signal through mucosal TGR5, which regulates smooth muscle contractility by complex mechanisms. Understanding how bile acids differentially regulate gastrointestinal motility could facilitate new therapeutic options for specific DGBIs.

## 1 Introduction

Disorders of gut-brain interaction (DGBIs) affect 40% of the global population and are more prevalent in women compared to men (1). Underlying many DGBIs is altered gastrointestinal motility (2). Numerous dietary, host, and microbial factors influence gastrointestinal motility. These include the bile acids, steroidal structures synthesized in the liver and modified by gut bacteria. Recent studies report altered bile acid homeostasis in a number of DGBIs. Decreased bile acid delivery to the colon is found in subsets of patients with constipation-predominant irritable bowel syndrome (IBS) (3). Conversely, many patients with diarrhea-predominant IBS have increased colonic bile acids (4). Mechanisms by which bile acids regulate gastrointestinal motility are incompletely understood.

Bile acids interact with ion channels and receptors in both the plasma membrane and nucleus in multiple cell types throughout the gastrointestinal tract (5). In the colon, pro-kinetic effects of bile acids are attributed to their interactions with the Takeda G protein-coupled receptor 5 (TGR5) (6), which is expressed in the plasma membrane of cells in the enteric ganglia, muscularis externa, and mucosa (7). TGR5 activation can induce numerous intracellular signaling pathways, including those mediated by the phosphorylation of extracellular signal-regulated kinase (ERK) (8). How these signaling cascades produce changes in smooth muscle cells remains unknown. One possibility is that bile acids directly activate TGR5 on smooth muscle cells; this possibility would require the bile acids to be actively transported across the enterocyte brush border membrane via the apical sodium-dependent bile acid transporter (ASBT) (9). In intestinal smooth muscle, contractility is regulated primarily through phosphorylation of myosin light chain (MLC), which facilitates cross-bridge formation and muscle contraction (10). It is not yet known whether contractile responses to bile acids in the ileum require TGR5 or ASBT, or whether they involve the phosphorylation of ERK or MLC.

One barrier to a more complete understanding of how bile acids regulate gastrointestinal motility is the limited number of *ex vivo* systems that model physiologic changes in motility resulting from bile acid exposure. Standard force-transduction assays suspend intact segments of intestine in organ baths with sutures that close off both ends of the segment, preventing large molecules like bile acids from accessing receptors on the mucosal surface. We recently validated a modified organ bath technique (Dike, Soni et al. under review) in which intact segments of mouse ileum are gently everted to fully expose the mucosal surface to the organ bath. In a screen of five abundant bile acids, we found that ursodeoxycholic acid (UDCA) stimulates longitudinal smooth muscle contractility in a dose-dependent manner, high physiologic concentration (100 μmol/L) of deoxycholic acid (DCA) inhibits contractility in ileum from female mice, but chenodeoxycholic acid (CDCA), glycocholic acid (GCA), and lithocholic acid (LCA) have no effect on contractility in everted mouse ileum.

This Brief Report aims to address four unanswered questions regarding the differential ileal contractile responses to UDCA and DCA. First, do these responses differ based on sex? Second, is the bile acid receptor TGR5 required? Third, is active transport of bile acids across the enterocyte brush border via ASBT required? Fourth, does mucosal application of bile acids promote phosphorylation of ERK and MLC? Answers to these questions will improve our understanding of the fundamental roles that UDCA and DCA play both in normal gastrointestinal physiology and in the pathogenesis of DGBIs.

## 2 Materials and methods

### 2.1 Animals

Male and female C57BL/6 J mice, originally sourced from Jackson Laboratory or Charles River, were obtained from the Center for Comparative Medicine at Baylor College of Medicine at 8-16 weeks of age and maintained on standard rodent chow (PicoLab Select Rodent 50 IF/6F; LabDiet). Animals were euthanized with isoflurane and cervical dislocation, and the distal small bowel was harvested for immediate study. The Institutional Animal Care and Use Committee at Baylor College of Medicine approved all aspects of this study.

### 2.2 Everted ileum preparations and contractility measurements

Everted ileum preparations and contractility measurements were performed as recently described (Dike, Soni et al. under review). Briefly, the most distal 10 cm of ileum just proximal to cecum was submerged in Krebs solution (mmol/L: 120.9 NaCl, 5.9 KCl, 2.5 CaCl_2_, 14.4 NaHCO_3_, 1.2 NaH_2_PO_4_, 1.2 MgCl_2_, 11.5 glucose), sutured to the notched end of a custom metal rod, and gently everted over the rod. The distal 5 mm segment containing the suture was removed and the everted tissue was removed from the rod. Full-thickness 1 cm everted segments were mounted in tissue baths filled with 25 mL Krebs solution, 250 μL 4% bovine serum albumin was added to prevent tissue swelling, and baths were perfused continuously with 95% O_2_/5% CO_2_ gas. Isometric force was measured by a force displacement transducer connected to a PowerLab recorder (ADInstruments). After 30 min of equilibration to 0.4 g tension, baseline activity was recorded for 5 min, then bile acid was added to the organ bath in increasing concentrations [250 μL UDCA (Cayman Chemicals # 15121) or DCA (MilliporeSigma # 30970) at doses of 0.1, 1, 10, and 100 μmol/L with stock solutions prepared in ethanol]. For inhibitor experiments, 125 μL of either GSK2330672 (ASBT inhibitor, Cayman Chemicals #23843) at 20 nM (11) or SBI-115 (TGR5 inhibitor, MedChemExpress # HY-111534) at 100 μM (12, 13) or DMSO buffer was added to the organ bath halfway through the equilibration period (at 15 min). To confirm adequate ASBT inhibition, the potent nuclear farnesoid-X-receptor (FXR) agonist bile acid chenodeoxycholic acid (CDCA, Millipore Sigma cat #220411) was added to tissue pretreated with GSK2330672 or buffer, and after 90 min at 0.4 g tension, tissue was removed, flash-frozen in liquid nitrogen, and stored at −80°C for transcriptional analysis of FXR target genes. For the experiments with UDCA and DCA, contractile activity was recorded for 5 min following each dose. After recordings were complete, intestinal length was measured and the tissue was removed, dried, and weighed. The amplitude and relative integral to minimum were normalized to tissue cross-sectional areas using the measured length and weight and an assumed tissue density of 1.05 g/cm^3^. Contractile magnitude was calculated as the mean delta force relative to baseline.

### 2.3 Western blot, RNA purification, and quantitative real-time PCR

Tissues were frozen in liquid nitrogen immediately after *ex vivo* contractility assays. Tissues were lyophilized, ground over liquid nitrogen, and suspended in RIPA buffer with 1x protease and phosphatase inhibitor mixture. Tissue suspensions were kept on a rotary shaker for 30 min in a cold room then were sonicated three times with 10 s pulses. Lysates were spun at 12,000 × g for 15 min and supernatants were saved. Anti-ERK 1/2 (Cell Signaling, Cat # 9102S), anti-MLC2 (Cell Signaling, Cat # 3672S), anti-pERK1/2 (Cell Signaling, Cat # 9101S), and anti-pMLC2 (S19) (Cell Signaling, Cat # 3671S) antibodies were used to detect total and phosphorylated ERK and MLC proteins. Alternatively, total RNA was isolated from frozen tissues using Direct-zol RNA Microprep Kit (Zymo Research, Cat # R2062) and quantified with a NanoDrop 2000c spectrophotometer (Thermo Fisher Scientific, Waltham, MA). Complementary DNA was synthesized from 1 μg RNA using iScript Reverse Transcription Supermix (Biorad, Hercule, CA, Cat # 1708840). SYBR Green PCR Master Mix (Thermo Fisher Scientific) was used on a StepOnePlus Real-Time PCR System (Applied Biosystems, Foster City, CA). Relative expression levels to β-actin were calculated by the comparative cycle threshold (ΔΔCt) method. The following primers were used: Fgf15 forward primer: 5’-AGGAGGACCAAAACGAACGAA-3’ Fgf15 reverse primer: 5’-GAGTAGCGAATCAGCCCGTAT-3′ β-actin forward primer: 5’-GCAGGAGTACGATGAGTCCG-3′ β-actin reverse primer: 5’-ACGCAGCTCAGTAACAGTCC -3’.

### 2.4 Statistical analyses

All analyses were performed using Prism 10 (GraphPad Software). Dose-dependent force-transduction data were analyzed by two-way repeated measures ANOVA using the method of Geisser and Greenhouse to correct for violations of the assumption of sphericity. When the global treatment/dose interaction effect was significant (*p* < 0.05), Sidak’s multiple comparisons tests were used to determine differences between groups for each dose of bile acid. Western blot and qPCR data were evaluated by *t*-test for comparisons between two groups or by ANOVA with Tukey’s multiple comparisons test to determine differences among three groups. Data are representative of at least two confirmatory experiments performed with separate sets of animals.

## 3 Results

### 3.1 Contractile responses to UDCA or DCA are not dependent on sex

**Figure 1:**
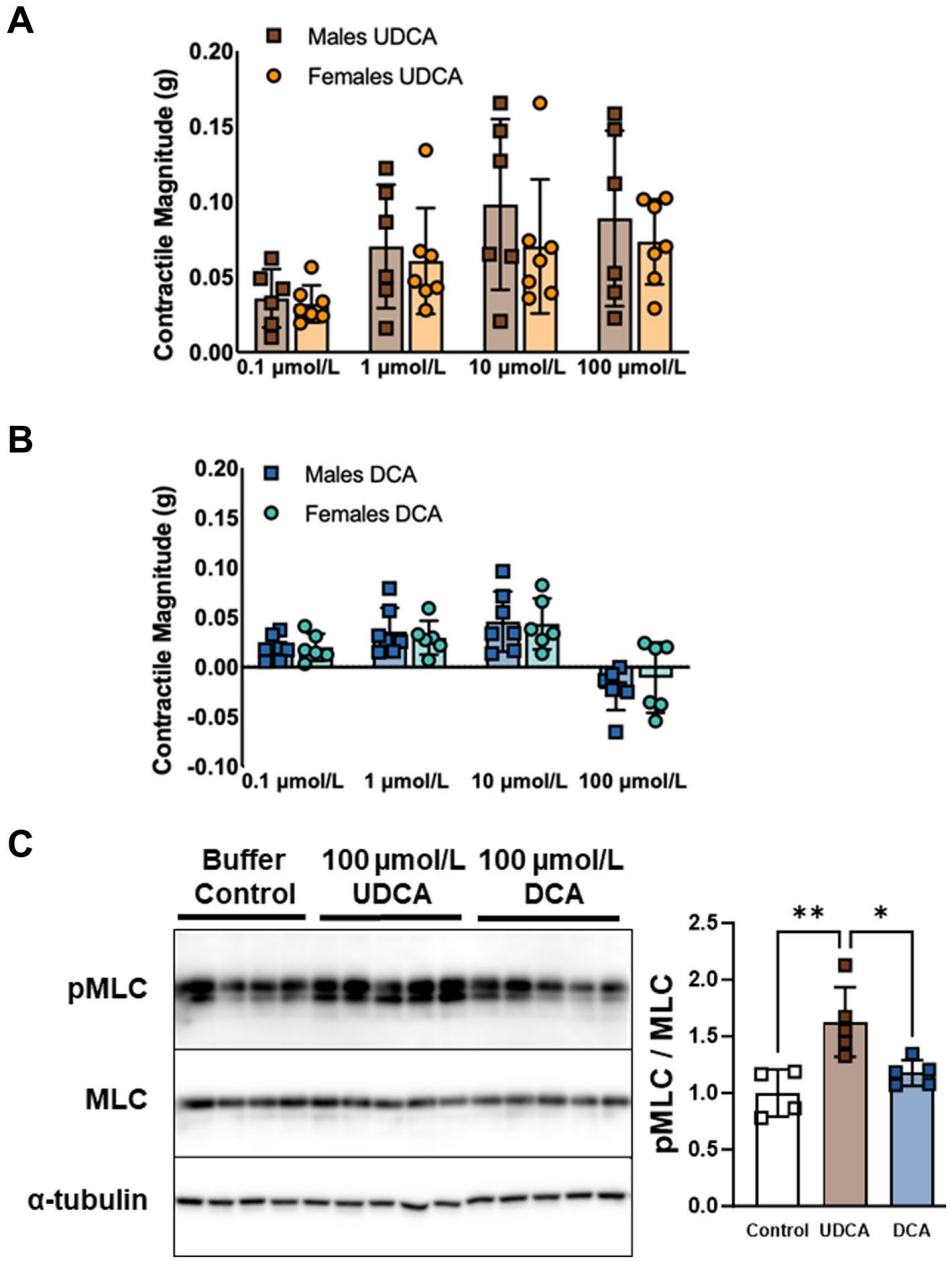
UDCA and DCA differentially affect ileal longitudinal smooth muscle contractility and MLC phosphorylation. **(A)** Dose-dependent contractile responses to UDCA are similar in everted ileum from male versus female mice. **(B)** DCA stimulates contractility at doses of 0.1, 1.0, and 10 μmol/L and inhibits contractility at 100 μmol/L in everted ileum similarly in male versus female mice. **(C)** MLC phosphorylation increases in response to 100 μmol/L UDCA but not DCA in ileum from male mice. Mean + SD; *n* = 4–7; **p < 0.01 and *p < 0.05. DCA, deoxycholic acid; MLC, total myosin light chain; pMLC, phosphorylated myosin light chain; UDCA, ursodeoxycholic acid.

Our modified tissue bath technique quantifies contractile responses to bile acids applied to everted segments of ileum. Previously, we tested five of the most abundant bile acids and found that neither CDCA, GCA, nor LCA affect contractility. However, UDCA and DCA stimulate contractility at low doses and have opposing effects at a high physiologic dose (100 μmol/L) (Dike, Soni et al. under review). Here, we sought to determine whether contractile responses to bile acids are dependent on sex. Confirming our previous results, female mouse ileum responded to UDCA with increases in contractile magnitude (Figure 1A) and responded modestly to DCA with increased contractility at doses of 0.1, 1.0, and 10 μmol/L and decreased contractility at 100 μmol/L (Figure 1B). Everted ileum from males responded identically to females to both UDCA (*p* = 0.46) and DCA (*p* = 0.54). In addition to contractile magnitude, we found no differences between males and females in the total activity, amplitude, or frequency of contractions (Supplementary Figure S1). We further examined male ileum with western blot and confirmed that 100 μmol/L of UDCA but not DCA increased phosphorylation of MLC (Figure 1C), in accord with increased contractility. These results confirm a lack of sex differences in the contractile responses to UDCA and DCA applied to the ileal mucosal surface, and that high-dose UDCA but not DCA promotes MLC phosphorylation and increased contractility.

### 3.2 Contractile responses to UDCA and DCA on ileal smooth muscle require TGR5

**Figure 2:**
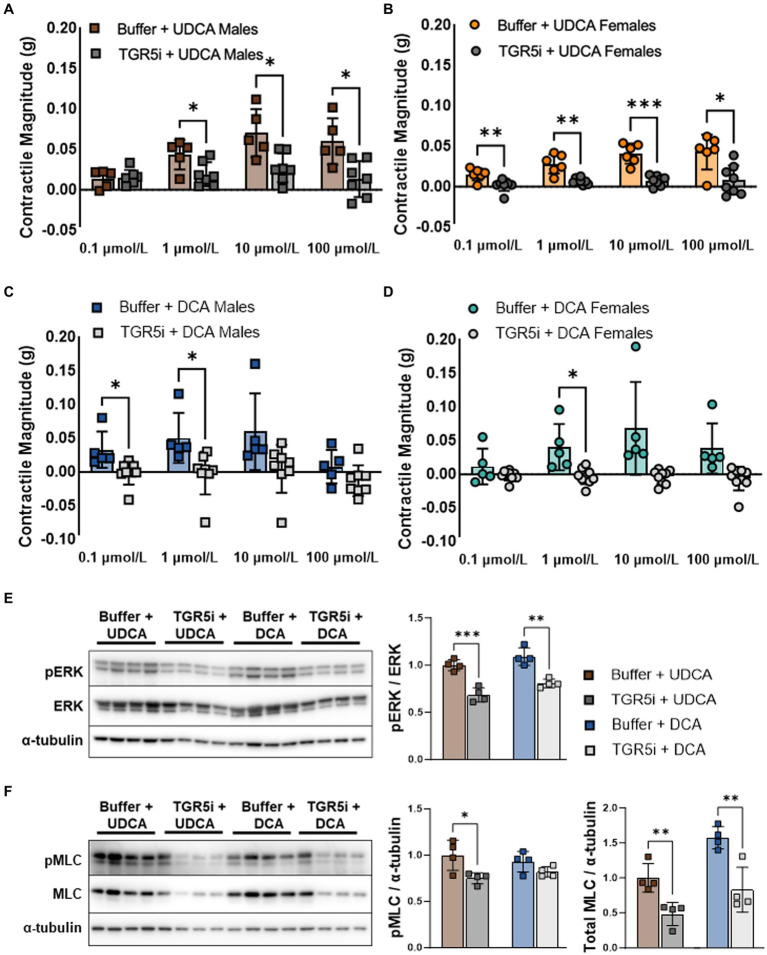
TGR5 inhibition minimizes contractile responses to both UDCA and DCA in everted ileum from male and female mice. **(A–D)** Contractility mediated by UDCA and low-dose DCA is blunted by pretreatment with TGR5i. **(E)** TGR5i pretreatment decreases ERK phosphorylation due to 100 μmol/L UDCA or DCA, consistent with blunted contractile responses. **(F)** TGR5i pretreatment unexpectedly decreases both phosphorylated and total MLC. Mean + SD; *n* = 4–6; ***p < 0.001, **p < 0.01, and *p < 0.05. DCA, deoxycholic acid; ERK, total extracellular signal-regulated kinase; pERK, phosphorylated extracellular signal-regulated kinases; pMLC, phosphorylated myosin light chain; MLC, total myosin light chain; TGR5i, Takeda G protein-coupled receptor 5 inhibitor SBI-115; UDCA, ursodeoxycholic acid.

In the colon, prokinetic responses to bile acids are mediated in part by the plasma membrane bile acid receptor TGR5 (6). TGR5 phosphorylates ERK among other kinases (8). We previously reported that TGR5 agonists stimulate ileal contractility similar to UDCA and low-dose DCA (Dike, Soni et al. under review). To confirm that bile acids increase smooth muscle contractility through TGR5, we pretreated everted ileum with the TGR5 inhibitor (TGR5i) SBI-115, then measured contractile responses to UDCA or DCA. Pretreatment with TGR5i minimized the contractile response to UDCA in both males (Figure 2A) and females (Figure 2B), and minimized the response to DCA in males (Figure 2C) and females (Figure 2D). Consistent with these results, TGR5i decreased ERK phosphorylation in male ileum exposed to 100 μmol/L of either bile acid (Figure 2E). TGR5i also decreased levels of phosphorylated MLC in bile acid treated ileum as expected; however, TGR5i did not decrease the ratio of phosphorylated MLC to total MLC and this was due to an unexpected decrease in total MLC levels (Figure 2F). Similar trends were observed when the tissue was stimulated with lower dose (10 μmol/L) UDCA or DCA (Supplementary Figure S3). Taken together, these data confirm that TGR5 activation mediates the contractile responses to UDCA and to low-dose DCA in the mouse ileum, and that TGR5 inhibition may decrease smooth muscle contractions through decreased MLC phosphorylation or by other means.

### 3.3 UDCA and DCA do not require ASBT to elicit contractile responses

**Figure 3:**
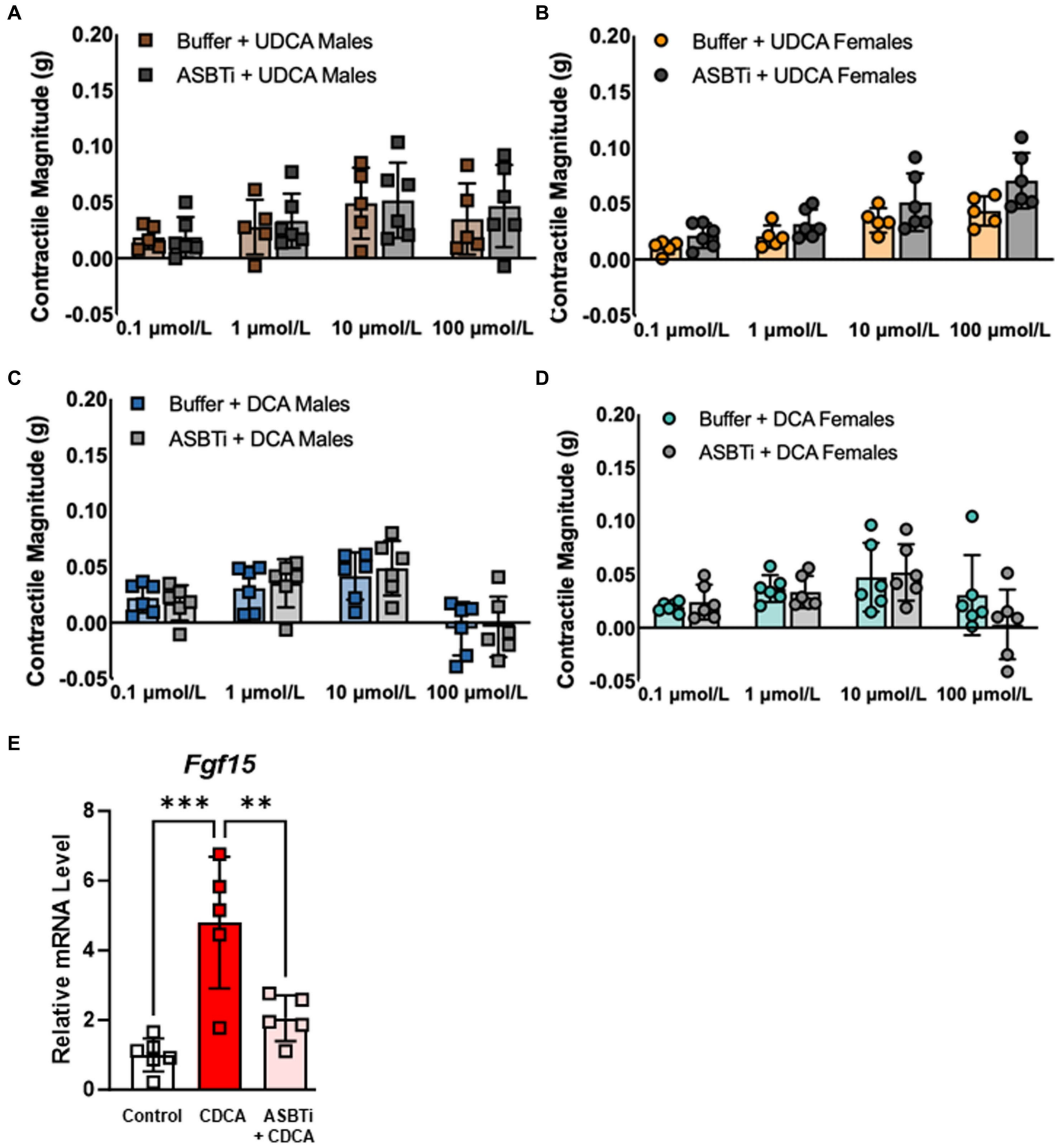
ASBT inhibition has no effect on contractile responses to either UDCA or DCA in everted ileum from male or female mice. **(A–D)** Contractility mediated by UDCA and low-dose DCA is not affected by pretreatment with ASBTi. **(E)** CDCA is taken up by ASBT and activates the nuclear bile acid receptor FXR, leading to transcriptional induction of its target gene *Fgf15*; this transcriptional response is blunted by pretreatment with ASBTi, confirming that bile acids are unable to enter cells. Mean + SD; *n* = 5–8; ***p < 0.001 and **p < 0.01. ASBTi, apical sodium-dependent bile acid transporter inhibitor GSK2330672; CDCA, chenodeoxycholic acid; DCA, deoxycholic acid; FXR, farnesoid-X-receptor; UDCA, ursodeoxycholic acid.

Because TGR5 is expressed by cells throughout the mucosa, muscularis externa, and enteric ganglia (7), we sought to determine whether bile acids require active transport across the enterocyte brush border in order to stimulate smooth muscle contractions. Pretreatment of everted ileum with the ASBT inhibitor (ASBTi) GSK2330672 did not alter contractile responses to UDCA in males (Figure 3A) or females (Figure 3B), and did not alter contractile responses to DCA in males (Figure 3C) or females (Figure 3D; see also Supplementary Figure S2). To confirm inhibition of intracellular bile acid uptake by ASBTi, everted ileum was treated with CDCA, a potent agonist of the nuclear bile acid receptor FXR, which led to transcriptional induction of the FXR target gene *Fgf15*. Pretreatment with ASBTi minimized this transcriptional response (Figure 3E), indicating that ASBTi prevents bile acids from crossing the brush border and activating their nuclear receptors. All together, our data suggest that bile acids signal through TGR5 on the ileal mucosal surface and do not require active transport across the brush border to promote MLC phosphorylation and smooth muscle contractility.

## 4 Discussion

We examined everted segments of mouse ileum using a modified tissue bath technique to show that 100 μmol/L UDCA but not DCA increases MLC phosphorylation and stimulates longitudinal smooth muscle contractility in male and female mice similarly. TGR5 inhibition minimizes contractile responses to either bile acid, and this is associated with decreased quantities of both phosphorylated and total MLC protein. On the other hand, ASBT inhibition does not affect contractile responses to UDCA or DCA, suggesting that bile acids mediate smooth muscle contractility by activating TGR5 on the mucosal surface rather than in submucosal compartments.

These data add to the emerging literature supporting the notion that bile acids differentially affect motility in various gastrointestinal regions (5). Prokinetic effects of UDCA are consistent with our prior studies with everted mouse ileum (Dike, Soni et al., under review). Likewise, inhibitory effects of DCA have been reported in non-everted segments of mouse ileum and colon in organ baths; one proposed mechanism of inhibition by DCA is via activation of TGR5 expressed on inhibitory motor neurons and descending interneurons that express nitric oxide synthase (6, 7). However, UDCA does not affect contractility in non-everted mouse colon *ex vivo* (6). This disagreement with our results could be due to intrinsic differences in signal transduction events initiated by bile acids in small bowel versus large bowel, or could be due to the lack of accessibility of bile acids to the mucosal surface in non-everted intestinal segments in isolated tissue bath assays. To help distinguish among these possibilities, one could measure contractile forces from bile acids applied to the apical surface of flat sheet intestine preparations or generate spatiotemporal maps of segments infused intraluminally with bile acids (14).

This study is the first to our knowledge to demonstrate that delivery of bile acids to the mucosal surface results in increased phosphorylation of MLC which is expressed in the muscularis externa. How enterocyte brush border TGR5 communicates with the muscularis externa is unclear, but active transport by ASBT is not essential. In CRE-driven luciferase reporter assays in TGR5-transfected Chinese hamster ovary cells, DCA (EC_50_ = 1.25) is 29-fold more potent than UDCA (EC_50_ = 36.4) (15). Other TGR5 agonists including CDCA and LCA (EC_50_ = 6.71 and 0.58) (15) do not modify contractile responses in everted mouse ileum, suggesting that the differential contractile responses to bile acids cannot be attributed to TGR5 binding affinity alone. Although inhibition of TGR5 blunts both ERK phosphorylation and contractile responses to UDCA and DCA, mechanisms underlying the selective decrease in total MLC protein in TGR5i-treated ileum segments are unclear and will be explored in future studies.

Although sex differences are well-documented in many DGBIs (1), in this study we did not identify any sex differences in ileal contractile responses to UDCA or DCA. Previously we reported subtle sex differences in upper gastrointestinal transit in post-pubertal mice (16). Our current data do not definitively exclude the possibility of sexual dimorphism in the ileal response to bile acids, given that our studies were limited by the large standard deviations observed in contractile responses. One potential source of variance is the broad age range (8–16 weeks) of mice used for these studies. Age-related changes in the enteric nervous system that impact intestinal motility are well documented and especially prominent later in life (17–21). However, dynamic changes that influence gastrointestinal motility also have been described from birth to young adulthood. Recently described examples include the GABA receptor system (22), dopaminergic signaling (23), and protein composition of smooth muscle (24). Similarly, cell composition changes dynamically in this early-life window. The early postnatal enteric nervous system is populated by neurons and glia derived from neural crest precursors, whereas by young adulthood mesoderm-derived enteric neurons have increased in abundance with potential impacts on intestinal motility (25). In addition to variable ages, the possibility of variable tissue damage during the eversion process also could contribute to the observed variance. Of note, our *ex vivo* model does not include a stress challenge, which is thought to contribute to many of the sexually dimorphic features of DGBIs (26).

In conclusion, the data presented in this Brief Report illustrate that UDCA and DCA differentially influence ileal smooth muscle contractility at physiologic concentrations, and we propose that mucosal TGR5 activation by bile acids ultimately affects MLC phosphorylation and longitudinal smooth muscle contractility. These effects are not dependent on transport across the brush border by ASBT or host sex. Ongoing investigations aim to determine the precise mechanisms of communication between enterocyte plasma membrane TGR5 and smooth muscle cells. Future studies also will explore a potential role of enteric neurons in regulating the bile acid response. Understanding how bile acids differentially affect intestinal motility will contribute to our understanding of the pathophysiology of the subset of DGBIs that are thought to be due to altered bile acid homeostasis, and might facilitate the development of more targeted therapeutic approaches for these challenging diagnoses.

## Data availability statement

The original contributions presented in the study are included in the article/Supplementary material, further inquiries can be directed to the corresponding author.

## Ethics statement

The animal study was approved by Baylor College of Medicine Institutional Animal Care and Use Committee. The study was conducted in accordance with the local legislation and institutional requirements.

## Author contributions

DC: Conceptualization, Data curation, Formal analysis, Investigation, Methodology, Visualization, Writing – review & editing, Validation, Writing – original draft. KS: Conceptualization, Data curation, Formal analysis, Investigation, Methodology, Validation, Visualization, Writing – review & editing, Project administration, Supervision. GP: Conceptualization, Data curation, Formal analysis, Investigation, Methodology, Project administration, Supervision, Visualization, Writing – review & editing, Funding acquisition, Resources.
