# Supplementary figures and images for: Smooth muscle contractile responses to bile acids in mouse ileum require TGR5 but not ASBT
Source: Front Neurol. 2024 Apr 24;15:1334319. doi: 10.3389/fneur.2024.1334319 (PMC11076673; doi:10.3389/fneur.2024.1334319)

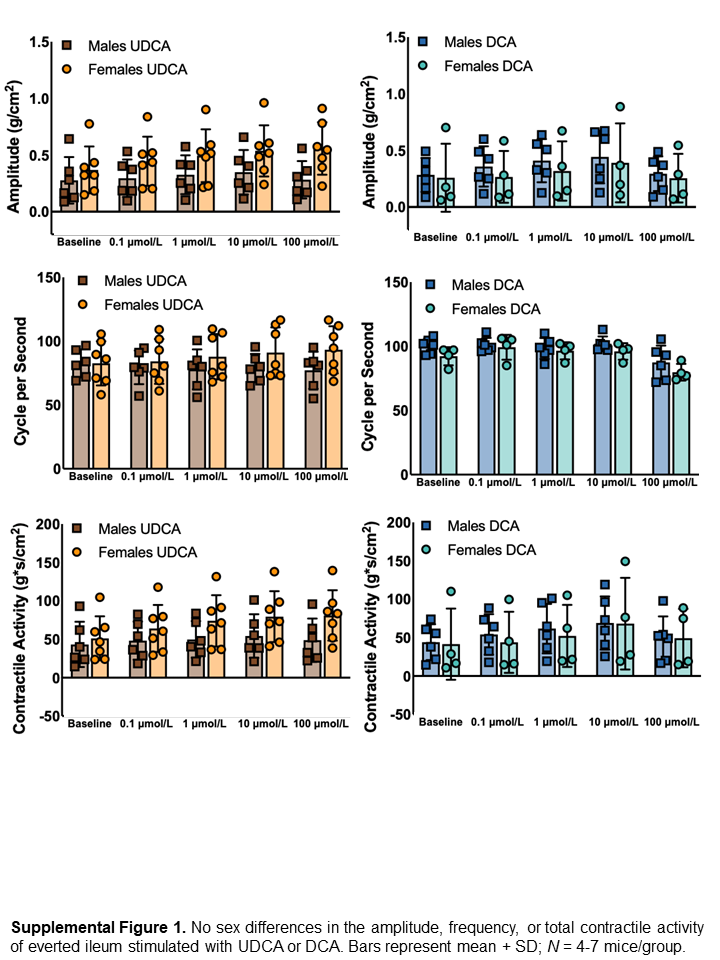


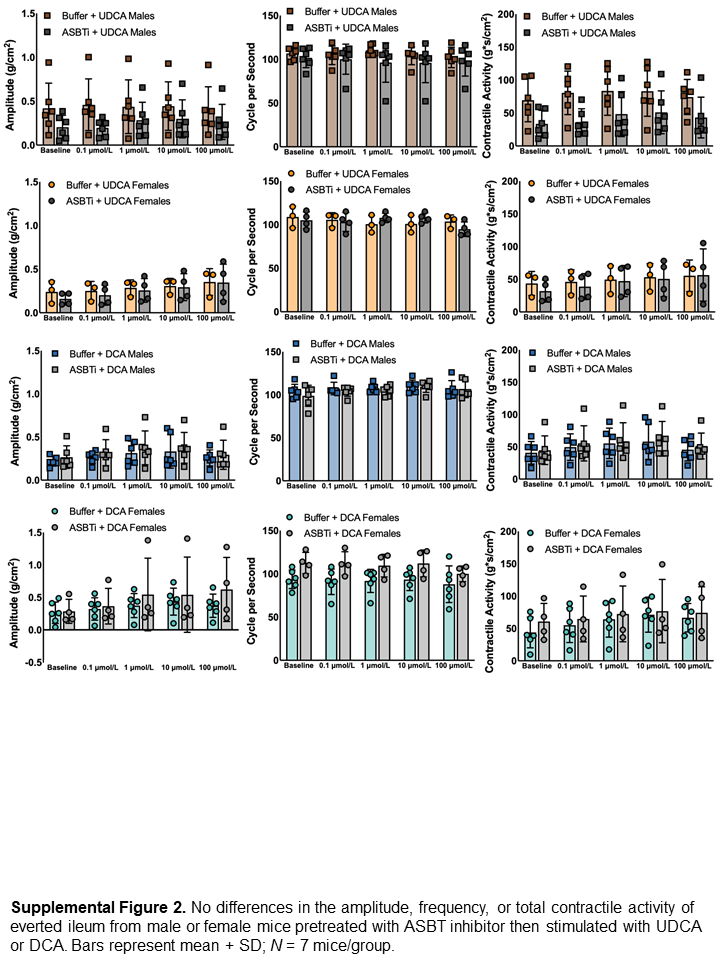


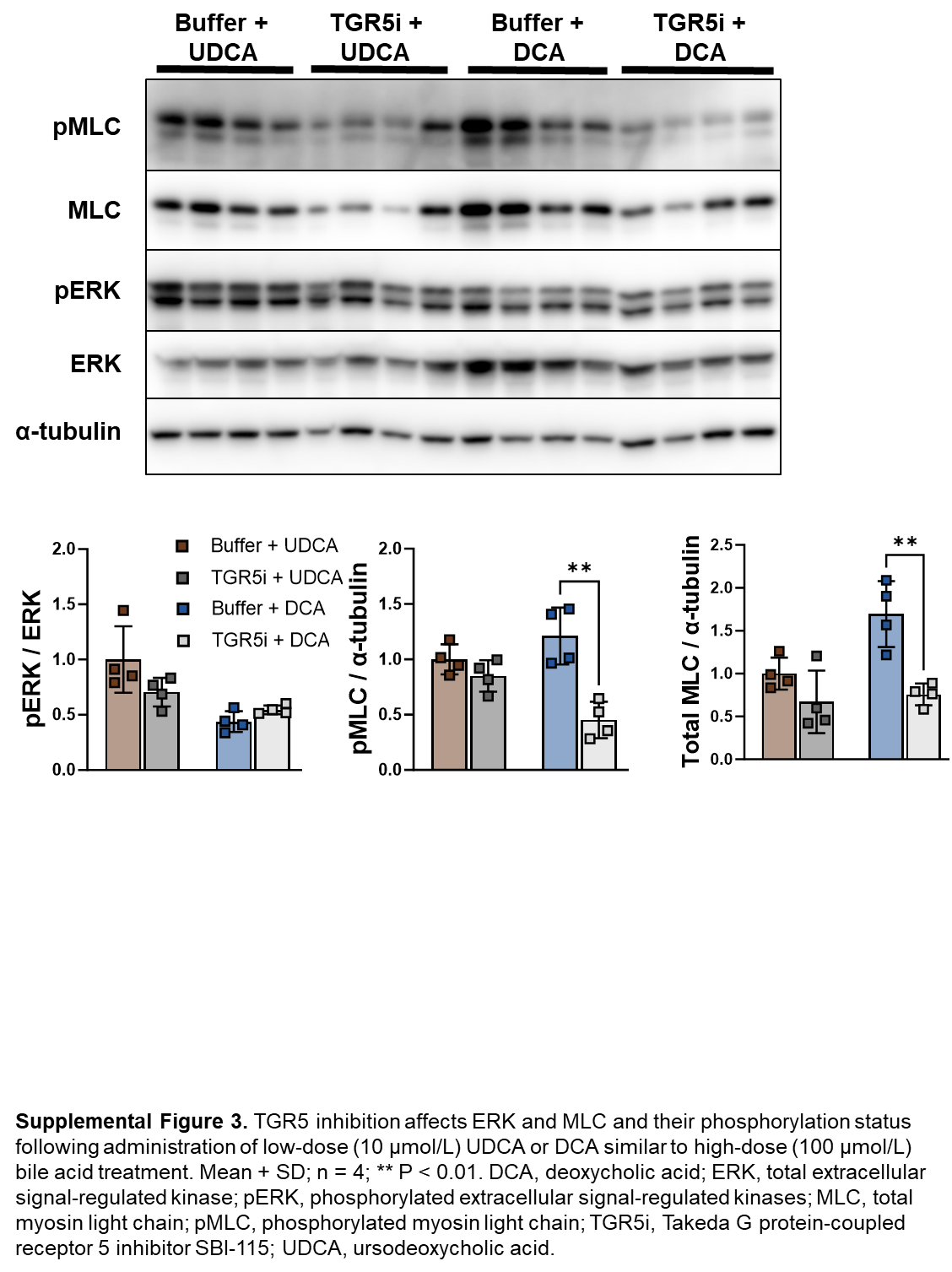

Supplement: Supplementary file 1 [file Table_1.DOCX]
